# Supplementary material for: Repurposing Hsp90 inhibitors as antimicrobials targeting two-component systems identifies compounds leading to loss of bacterial membrane integrity
Source: Microbiol Spectr. 2024 Jun 25;12(8):e00146-24. doi: 10.1128/spectrum.00146-24 (PMC11302729; doi:10.1128/spectrum.00146-24)
Supplement: Supplemental material — Fig. S1 to S5; Tables S1 to S4. [file spectrum.00146-24-s0001.docx]

**Supplemental material**


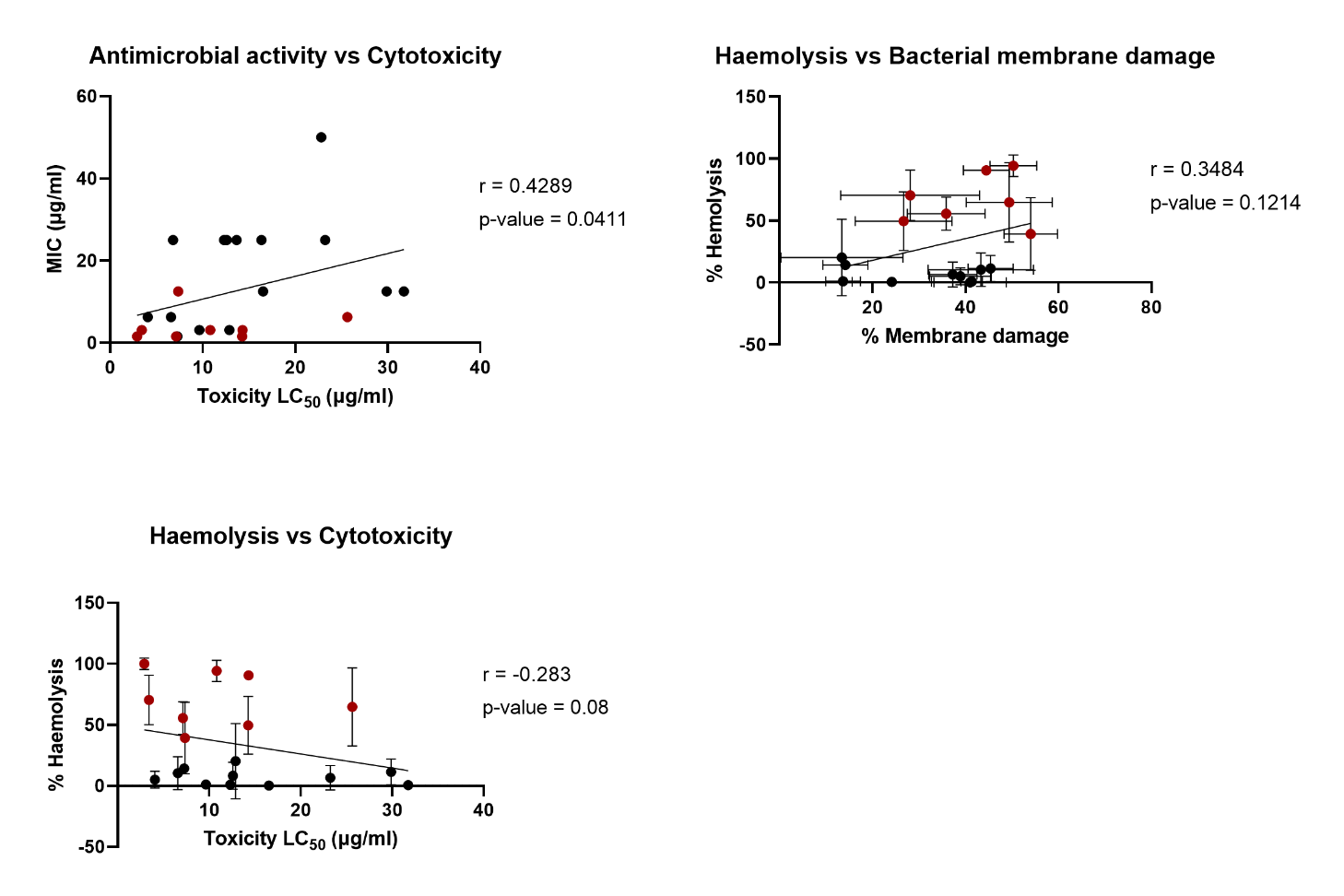


**Supplementary Figure SF1. Correlation graphs of relevant parameters, including Spearman rank-correlation coefiecient (r) and p-value. A.** Correlation between the antimicrobial activity expressed in MIC (μg/ml) and toxicity expressed as LC_50_ (μg/ml). **B.** Correlation between haemolysis percentage in sheep red blood cells and percentage of membrane damage (Suplementary Table 3). **C**. Correlation between percentage of haemolysis in sheep red blood cells and toxicity expressed as LC_50_ (μg/ml). Haemolytic compounds are shown in red.

**Table ST1. MIC data of DPP compounds against a panel of Gram-positive and Gram-negative strains, including *E. coli* outer membrane and efflux mutants.** n.t. non-tested, *E. coli* JW5503 (Δ*tolC* mutant), *E. coli* D21f2 defective LPS core..

| **DPP comp.** |  |  | **MIC (μg/ml)** | | |  |  |
| --- | --- | --- | --- | --- | --- | --- | --- |
|  | ***E. faecium*** | ***E. faecalis*** | ***P. haemolytica*** | ***P. aeruginosa*** | ***E. coli*** | ***E. coli***  **JW5503** | ***E coli* D21f2** |
| **1** | 12.5 | 12.5 | 25 | >250 | >250 | 1.56 | n.t. |
| **2** | 25 | 25 | 50 | >250 | >250 | 6.25 | 50 |
| **3** | >250 | >250 | 50 | >250 | >250 | 1.56 | n.t. |
| **4** | 3.12 | 3.12 | 125 | >250 | >250 | 6.25 | >50 |
| **5** | 12.5 | 12.5 | 12.5 | >250 | 50 | 3.12 | 50 |
| **6** | 6.25 | 6.25 | 12.5 | >250 | >250 | 3.12 | 25 |
| **7** | 3.12 | 3.12 | 25 | >250 | >250 | 3.12 | 25 |
| **8** | 3.12 | 1.56 | 250 | >250 | >250 | 12.5 | 25 |
| **9** | 25 | 25 | 25 | >250 | >250 | 12.5 | 50 |
| **10** | 250 | 250 | 62.5 | >250 | >250 | 6.25 | 250 |
| **11** | 12.5 | 12.5 | 62.5 | >250 | >250 | 1.56 | 25 |
| **12** | 50 | 50 | 62.5 | >250 | >250 | 12.5 | >50 |
| **13** | 25 | 25 | >250 | >250 | >250 | 12.5 | >50 |
| **14** | 12.5 | 12.5 | >250 | >250 | >250 | 3.13 | n.t. |
| **15** | 1.56 | 1.56 | >250 | >250 | >250 | 3.12 | n.t. |
| **16** | 50 | 50 | 50 | >250 | >250 | 3.13 | n.t. |
| **17** | >250 | 250> | 50 | >250 | >250 | 6.25 | n.t. |
| **18** | 50 | 50 | >250 | >250 | >250 | 6.25 | n.t. |
| **19** | 50 | 50 | >250 | >250 | >250 | 12.5 | n.t. |
| **20** | 25 | 25 | >250 | >250 | >250 | 6.25 | n.t. |
| **21** | 12.5 | 12.5 | 12.5 | >250 | >250 | 1.56 | >250 |
| **22** | 12.5 | 12.5 | 12.5 | >250 | >250 | 1.56 | 25 |
| **23** | 25 | 12.5 | 31.2 | >250 | 125 | 25 | 50 |
| **24** | >250 | >250 | 31.2 | >250 | 125 | 25 | 50 |


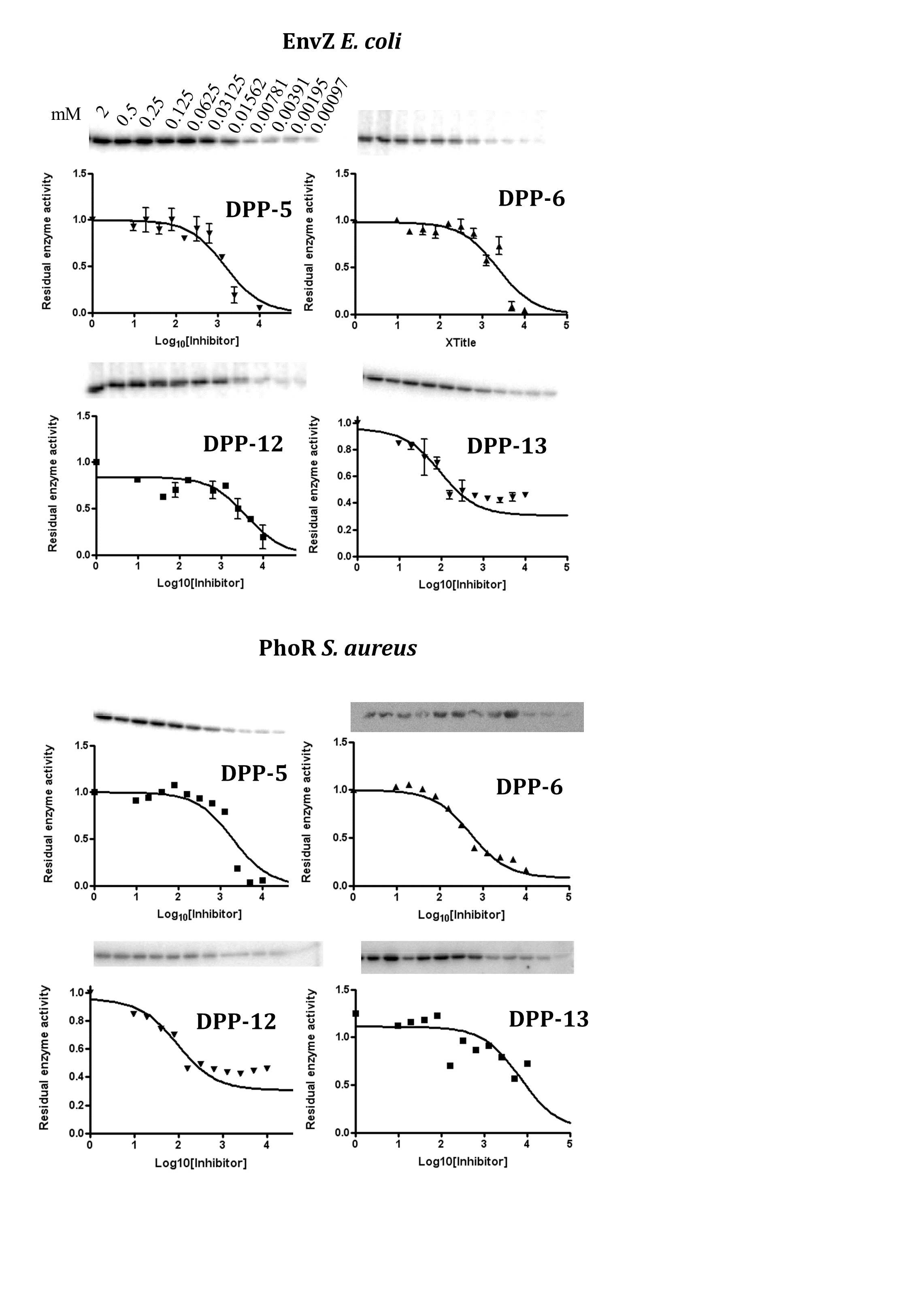


**Figure SF2. Autophosphorylation inhibition assays of DPP compounds against EnvZ form *E. coli* and PhoR from *S. aureus.*** A range of concentrations of DPP compounds was tested for inhibition of autophosphorylation of two HKs (PhoR from *S. aureus* and EnvZ from *E. coli).* Amount of phosphorylated HK was detected via Western Blot and percentage of autophosphorylation calculated according to the positive (ATP) and inhibition (ATP+AMP-PNP) controls. Curves were plotted using Prism 4.1.


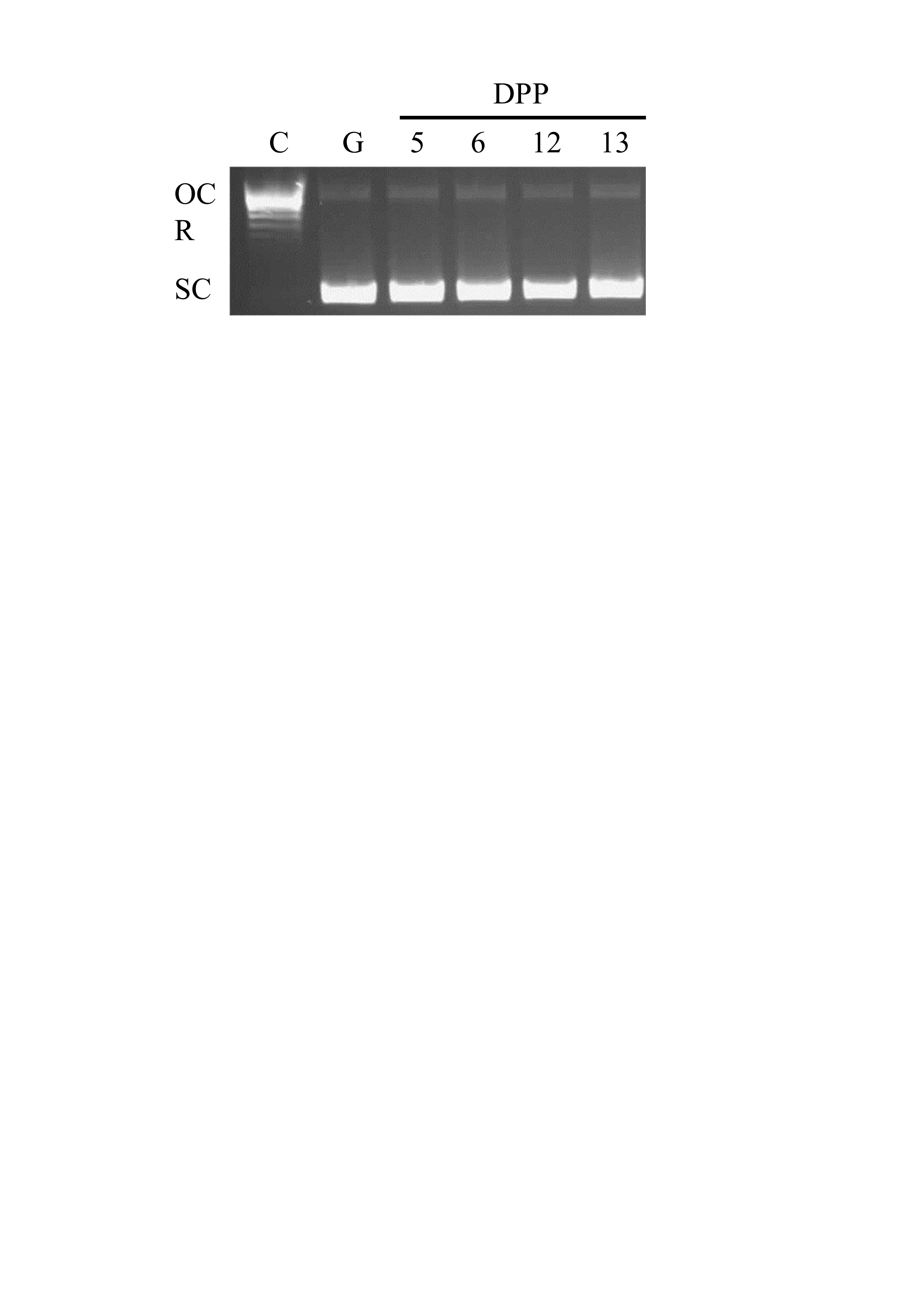


**Figure SF3. Bacterial gyrase inhibition assay of DPP compounds.** Gyrase inhibition was tested using a supercoiling inhibition assay. Control (C) contained relaxed pBR322, Gyrase control (G) contains relaxed pBR322 and *E. coli* gyrase (0.1 mU/reaction). DPP-compounds were added at a concentration of 2 mM. No inhibition of the supercoiled activity of *E. coli* gyrase is observed by any of the DPP compounds. OC = nicked, open circular; R = relaxed topoisomers; SC = supercoiled topoisomers

**Supplementary method**

**Gyrase inhibition assay.** Gyrase activity inhibition assays were performed using *E. coli* gyrase supercoiling kit (Inspiralis, Norwich, UK)(66) following the manufacturer instructions. Briefly, assay buffer (35 mM Tris-HCl pH 7.5, 24 mM KCl, 4 mM MgCl_2_, 2 mM DTT, 1.8 mM spermidine, 1 mM ATP, 6.5% (w/v) glycerol, and 0.1 mg/ml albumin) was mixed with 0.5 μg relaxed pBR322, and 2 mM final concentration of desired inhibitor in DMSO. Reaction was started by adding 0.1 mU of *E. coli* gyrase per reaction, for a total final volume of 30 μl per reaction. Samples were incubated for 30 mins at 37 ^o^C, stopped by adding 30 μl STEB (40% sucrose, 100 mM Tris-HCl pH 8.0, 10 mM EDTA and 0.5 mg/ml bromophenol blue) and 30 μl 24:1 chloroform/isoamyl alcohol and mixed by vortexing. Samples were centrifuged for 1 minute and 30 μl was loaded into a 1% agarose gel supplemented with SYBRSafe^TM^ followed by electrophoresis at 85 V for 2 hours. Gels were visualized using GelDoc XR+ gel documentation system (Bio-Rad).

| **Treatment** | **% FITC stained bacteria 0.5 xMIC** | **% FITC stained bacteria 1 xMIC** | **% FITC stained bacteria 2 xMIC** |
| --- | --- | --- | --- |
| Unstained | 0 | | |
| Alive | 4.10 | | |
| Nisin | 16.37 | 53.32 | 59.41 |
| Novobiocin | 8.28 | 12.62 | 13.17 |
| 1 | 30.46 | 39.72 | 46.63 |
| 2 | 49.81 | 54.03 | 63.56 |
| 3 | 35.78 | 49.41 | 58.28 |
| 4 | 7.94 | 14.15 | 19.78 |
| 5 | 13.40 | 13.42 | 41.29 |
| 6 | 36.93 | 44.48 | 48.91 |
| 7 | 42.44 | 50.30 | 54.59 |
| 8 | 12.26 | 32.15 | 36.10 |
| 9 | 38.22 | 41.34 | 43.78 |
| 10 | 28.57 | 37.28 | 40.99 |
| 11 | 24.77 | 28.12 | 57.97 |
| 12 | 9.04 | 24.16 | 29.11 |
| 13 | 35.10 | 45.40 | 45.44 |
| 14 | 28.78 | 44.58 | 56.62 |
| 15 | 9.29 | 13.68 | 18.42 |
| 20 | 31.90 | 40.99 | 50.97 |
| 21 | 31.51 | 35.84 | 51.03 |

**Suplementary Table ST2. Porcentage of FITC-stained bacteria as measured using flow-citometry at different concentrations and different treatments.**

**Table ST3. Overview of the data obtained for the DPP compounds.** The overview includes compound structure, MIC against *S. aureus* (in μg/ml and μM), LC_50_ in HEK293 cells (in μg/ml and μM), selectivity index (LC_50_/MIC), inhibition of PhoR histidine kinase (μM), inhibition of Hsp90 (IC_50_ in μM), if it is considered to cause membrane damage to *S. aureus* and if it is considered to cause haemolysis to red blood cells (red)*. n.d. not done; N.A. not applicable.*

| **DPP comp.** | **X** | **R_1_** | **R_2_** | **R_3_** | **R_4_** | **R_5_** | **MIC S. aureus** | **LC_50_ HEK293 cells**  **μg/ml (μM)** | **Selectivity Index** | **Inhibition HKs** | **Membrane damage** | **Haemolysis** | **Inhibition Hsp90** |
| --- | --- | --- | --- | --- | --- | --- | --- | --- | --- | --- | --- | --- | --- |
| **1** | N | -CF_3_ | -OH | -H | -H | -H | 6.25 (19.53) | 4.06 (12.69) | 0.65 |  | YES | NO |  |
| **2** | N | -CF_3_ | -OMe | -H | -H | -H | 12.50 (37.3)† | 7.34 (21.91) | 0.60 | <2 mM | YES | MILD |  |
| **3** | N | -CF_3_ | -OEt | -H | -H | -H | 6.25 (17.86) | 25.64 (76.53) | 4.1 |  | YES | YES |  |
| **4** | N | -CF_3_ | -OBn | -H | -H | -H | 1.56 (3.80)† | 7.26 (17.71) | 4.65 |  | NO | NO |  |
| **5** | N | -CF_3_ | -OH | -H | -Cl | -H | 3.12 (8.79) | 12.88 (36.28) | 4.13 | 95 | MILD | NO | 0.496 |
| **6** | N | -CF_3_ | -OMe | -H | -Cl | -H | 3.12 (8.43)† | 14.30 (38.65) | 4.58 | 55 | YES | YES | 0.798 |
| **7** | N | -CF_3_ | -OEt | -H | -Cl | -H | 3.12 (8.21) | 10.82 (28.47) | 3.47 | <2 | YES | YES |  |
| **8** | N | -CF_3_ | -OBn | -H | -Cl | -H | 1.56 (3.51)† | 14.25 (32) | 9.14 |  | YES | YES |  |
| **9** | N | -Me | -OH | -H | -Cl | -H | 25 (83.33)† | 12.30 (41) | 0.49 |  | YES | NO |  |
| **10** | N | -Me | -OMe | -H | -Cl | -H | 25 (79.37) | 23.23 (73.74) | 0.93 |  | YES | NO |  |
| **11** | O | -CF_3_ | -OH | -H | -Cl | -H | 3.12 (8.76)† | 3.41 (9.61) | 1.10 |  | YES | YES |  |
| **12** | N | -CF_3_ | -OH | -H | -OMe | -H | 12.50 (35.71) | 31.74 (90.69) | 2.54 | 328 | MILD | NO | 0.574 |
| **13** | N | -CF_3_ | -OMe | -H | -OMe | -H | 12.50 (34.34)† | 29.88 (82.09) | 2.39 | 89 | YES | NO | 0.838 |
| **14** | N | -CF_3_ | -OEt | -H | -OMe | -H | 6.25 (16.45) | 6.57 (17.29) | 1.05 |  | YES | NO |  |
| **15** | N | -CF_3_ | -OBn | -H | -OMe | -H | 3.12 (7.09) | 9.64 (21.91) | 3.10 |  | NO | NO |  |
| **16** | N | -CF_3_ | -OH | -OMe | -H | -H | 25 (71.43) | 16.35 (46.71) | 0.65 |  | n.d. | NO |  |
| **17** | N | -CF_3_ | -OMe | -OMe | -H | -H | 25 (68.49) | 12.58 (34.47) | 0.50 |  | n.d. | NO |  |
| **18** | N | -CF_3_ | -OH | -H | -OMe | -OMe | 50 (131.58) | 22.81 (60.03) | 0.45 |  | n.d. | n.d. |  |
| **19** | N | -CF_3_ | -OMe | -H | -OMe | -OMe | 25 (63.29) | 13.65 (34.56) | 0.55 |  | n.d. | n.d. |  |
| **20** | N | -CF_3_ | -OEt | -H | -OMe | -OMe | 12.5 (30.49) | 16.53 (40.32) | 1.32 |  | YES | NO |  |
| **21*** | N | -CF_3_ | -H | -H | -Cl | -H | 1.56 (4.8)† | 7.12 (21.91) | 4.56 |  | YES | YES |  |
| **22** | N | -CF_3_ | -H | -H | -Cl | -H | 1.56 (4.59) | 2.89 (8.5) | 1.85 |  | n.d. | YES |  |
| **23**** | N | -CF_3_ | -OH | -H | -H | -H | 250 (961.54)† | >250 (>650) | N.A. |  | n.d. | n.d. |  |
| **24**** | N | -CF_3_ | -OMe | -H | -H | -H | 25 (102.04)† | 6.79 (27.71) | 0.27 |  | n.d. | n.d. |  |


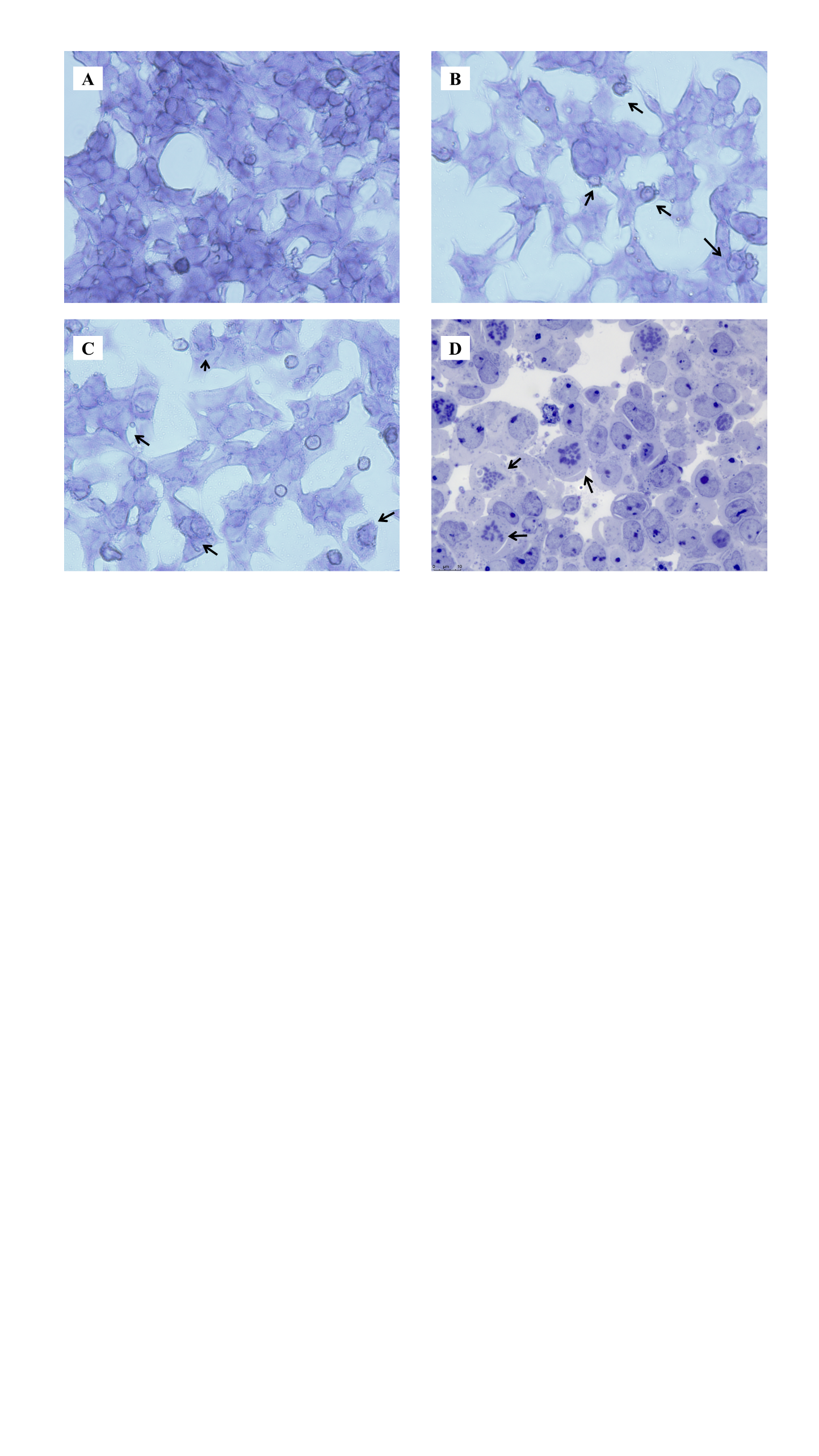


**Supplementary Figure SF4. Cell imaging of HEK293 cells after 24 hours exposure to DPP compounds.** A. HEK293 cells grown in exposure media B. HEK293 cells grown in the presence of LC_20_ concentration of **DPP-5** (5 μg/ml) (B), or **DPP-6** (6 μg/ml) (C) and imaged in 24-well plates at 40x amplification. Exposed cells were also transferred from the plate to a glass slide for visualization at 100x amplification of cells grown in the presence of LC_20_ (3 μg/ml) **DPP-14**. Arrows point to morphological abnormalities.

**Figure SF5. Schematic synthesis of DPP-22.**

**Table ST4. Data collection and refinement statistics for CheA-resorcinol complex.**

| **Data collection statistics** | **CheA-Resorcinol** |
| --- | --- |
| Beamline | ALBA-XALOC |
| Wavelength (Å) | 0.9795 |
| Resolution range (Å)* | 44.19-2.1 (2.17 -2.1) |
| Space group | P 2_1_ |
| Unit cell parameters  a, b, c (Å)  α, β, γ (°) | 40.97, 59.12, 67.06  90, 97.28, 90 |
| Total no. of reflections | 123148 (12471) |
| Unique reflections | 18443 (1847) |
| Completeness (%) | 98.42 (97.75) |
| Multiplicity | 6.7 (6.7) |
| Mean I/sigma(I) | 9.26 (3.56) |
| R_merge_ | 0.1321 (0.87) |
| R_pim_ | 0.05581 (0.3777) |
| R_work_ | 0.1867 (0.2102) |
| R_free_ | 0.2404 (0.2558) |
| No. of atoms  Protein  Water  Ligand | 2724  2680  28  16 |
| Rmsd:  Bonds (Å)  Angles (°) | 0.016  2.21 |
| Ramachandran plot (%)  Favored  Allowed  Outliers | 97.63  2.37  0.00 |
| Rotamer outliers (%) | 4.05 |
| Clashscore | 5.52 |
| B Factors (Å2)  Average  Macromolecules  Ligands  Solvent | 41.53  41.56  36.09  42.22 |
| Number of TLS groups | 2 |
| PDB code | 8PF2 |

*Values in parentheses are for the high-resolution shell.
